# Systematic gravity-induced posterior tumour drift in free-breathing lung stereotactic ablative radiotherapy

**DOI:** 10.1016/j.phro.2026.101030

**Published:** 2026-07-06

**Authors:** Lars Merring-Mikkelsen, Hella M.B. Sand, Mads H. Brincker

**Affiliations:** a Department of Medical Physics, Oncology, Aalborg University Hospital, Aalborg, Denmark; b Department of Clinical Medicine, Oncology, Aalborg University Hospital, Aalborg, Denmark

**Keywords:** Stereotactic ablative radiotherapy, Lung cancer, Intra-fractional tumour drift, Gravity-induced motion, Cone beam computed tomography, Free-breathing radiotherapy

## Abstract

**Background and purpose:**

Intra-fractional respiratory tumour motion is a known challenge in lung stereotactic ablative radiotherapy (SABR), as it can compromise target dose coverage. Gravity-induced tumour baseline drift during free-breathing carries the same risk but remains uncharacterised. This study aimed to quantify posterior tumour drift in peripheral lung SABR, investigate predictive factors, and evaluate tumour dose coverage.

**Materials and methods:**

In total, 97 patients (102 tumours) treated with 67.5 Gy in 3 fractions were analysed. Pre- and post-treatment cone beam computed tomography (CBCT) imaging quantified intra-fractional shifts at the first and second fraction. Temporal linear regression determined patient-specific drift rates. Correlations between drift rates and patient and tumour parameters were assessed. Plans were recalculated for worst-case drifts exceeding the 4 mm posterior planning target volume (PTV) margin.

**Results:**

Systematic posterior drift was observed, with mean vertical shifts of −1.45 mm at the first fraction and − 1.24 mm at the second fraction (both *p* < 0.001). Mean lateral and longitudinal shifts were negligible. No correlations with patient or tumour characteristics were identified. Based on drift rates, 15.7% of patients would exceed the 4 mm posterior margin within a 20-min treatment. Recalculated plans revealed severe gross tumour volume (GTV) underdosage: mean D_90%_ coverage decreased from 99.9% to 74.2% (*p* < 0.001) under worst-case scenarios.

**Conclusions:**

Gravity-induced posterior drift represents a systematic, clinically significant phenomenon in free-breathing lung SABR. In 15.7% of patients, drifts exceeded planning margins, with potentially severe target underdosage. Routine post-treatment CBCT verification is recommended to identify at-risk patients, enabling intervention at subsequent fractions to ensure treatment accuracy.

## Highlights

• Consistent tumour drift induced by gravity during lung radiotherapy treatment.
• Within 20 min 15.7% of tumours would exceed a 4 mm posterior margin.
• Posterior drift averaged −1.45 mm at fraction one and − 1.24 mm at fraction two.
• No patient or tumour specific factors predicted drift magnitude.
• Worst-case target coverage dropped from 99.9% to 74.2% for affected patients.

## Graphical abstract

Graphical abstract

**Figure:**
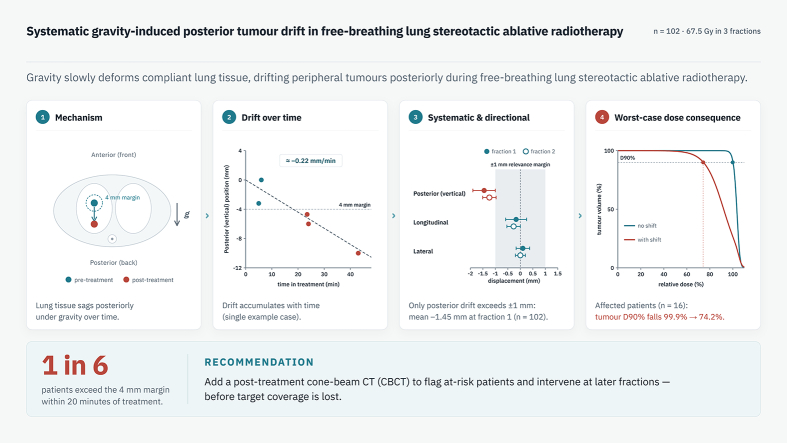

## 1 Introduction

Stereotactic ablative body radiotherapy (SABR) is a treatment modality that delivers high radiation doses to well-defined tumour targets while minimizing exposure to surrounding healthy tissue [1], [2]. Due to steep dose gradients and limited treatment margins inherent in SABR, even minor positional deviations can result in significant tumour underdosing or overdosing of adjacent organs at risk (OAR) [3], [4]. While SABR has become increasingly popular for early-stage non-small-cell lung cancer due to its efficacy and availability [5], ensuring positional accuracy throughout treatment delivery remains critical for both treatment success and patient safety. At our institution, clinically significant intra-fractional posterior tumour drifts have been observed for several peripheral lung SABR patients treated in free breathing while lying in a supine position, leading to implementation of a clinical workflow to manage this phenomenon.

Intra-fractional tumour motion during lung SABR is well-recognised as a major challenge to treatment precision. Respiratory motion is routinely managed using four-dimensional computed tomography (4DCT) to capture tumour position across breathing phases [6]. However, evidence shows that tumours can also exhibit baseline drifts independent of respiratory motion [7]. In gated lung SABR treatments with real-time tracking, cranial and posterior baseline drifts have been documented, with gravity mentioned as a potential contributing factor, though the underlying mechanisms have not been systematically investigated [8], [9]. Similarly, investigations using cone beam computed tomography (CBCT) of free-breathing lung SABR have reported intra-fractional tumour displacements with a small systematic posterior tendency, though the underlying mechanisms were not the primary focus of this study [10]. Recent European Society for Radiotherapy and Oncology (ESTRO) Advisory Committee on Radiation Oncology Practice (ACROP) guidelines acknowledge relaxation and gravity as potential sources of intra-fraction variation. However, they note the limited robust supporting evidence, highlighting the need for more systematic investigation [11].

To our knowledge, gravity-induced posterior tumour drift has not been systematically documented or quantified for lung SABR patients treated in free breathing. Posterior drift could potentially result from viscoelastic deformation of compliant lung parenchyma under constant gravitational loading. Physiological evidence supports this mechanism: gravity redistributes stress within lung tissue differently across body positions [12], [13], and lung parenchyma exhibits viscoelastic properties that lead to a time-dependent deformation under sustained loading [14]. Once a patient is positioned supine and immobilized, gravity may induce a slow, time-dependent posterior displacement as lung tissue gradually deforms under its own weight. This process is mechanistically distinct from the cyclic, reversible motion of respiration.

In this study, posterior tumour drift was systematically investigated in free-breathing peripheral lung SABR patients. Using repeated pre- and post-treatment CBCT imaging combined with a dedicated gravity-adapted workflow, intra-fractional tumour drifts were quantified, their temporal dynamics assessed, and potential correlations with patient- and tumour-specific parameters evaluated. Finally, the consequences for tumour dose coverage were analysed for clinically significant tumour drifts to determine their potential impact on treatment precision.

## 2 Materials and methods

### 2.1 Study design and patients

This study included 97 peripheral lung SABR patients with a total of 102 tumours (five patients had multiple tumours) treated between May 2024 and May 2025. Peripheral lung tumours were defined as lesions located >2.5 cm from the oesophagus, trachea, and bronchus, and > 0.5 cm from the aorta, heart, and spinal cord, in accordance with the Danish Lung Cancer Group (DOLG) criteria. No patients were excluded from the analysis. All patients received 67.5 Gy in 3 fractions and were treated in free breathing. Patients were immobilized using a lung board for the head and arms, combined with a Vac-Lok bag for the torso and a pelvic positioning board for the lower body.

Ethical approval for data collection and analysis was granted by the North Denmark Region Ethical Review Board (ID: K2024–215).

### 2.2 Treatment planning and delivery

All patients underwent a planning 4DCT consisting of 10 breathing phases, using a SOMATOM go.Open Pro CT scanner (Siemens Healthineers, Erlangen, Germany). The Gross Tumour Volume (GTV) was delineated on the mid-ventilation phase of the 4DCT and subsequently accumulated with the tumour positions from the remaining breathing phases to create an internal GTV (IGTV). Treatment plans were created using Eclipse v18.0.1 (Varian Medical Systems, Palo Alto, CA, USA) with a planning target volume (PTV) margin of 4 mm axially and 5 mm craniocaudally from the IGTV. The beam arrangements consisted of static open fields, typically seven coplanar beams, distributed as an arc over the hemithorax containing the tumour. Beam angles were selected individually to optimize target coverage while minimizing dose to OAR. Treatment was delivered using 6 MV flattening-filter-free beams with a dose rate of 1400 monitor units/min on a TrueBeam linear accelerator (Varian Medical Systems, Palo Alto, CA, USA). During treatment, a dedicated gravity-adapted workflow was utilised.

### 2.3 Gravity-adapted workflow

At the first fraction (F1), two CBCT scans were systematically acquired, a pre-treatment CBCT_1_ (pre-CBCT_1_) for bone and tumour matching followed by treatment delivery, and a post-treatment CBCT (post-CBCT) for offline analysis. During online matching the vertical difference in tumour position relative to bony anatomy between planning CT and CBCT (Δ_vrt_) were evaluated. If Δ_vrt_ exceeded 5 mm in pre-CBCT_1_, an additional pre-CBCT_2_ was acquired after a waiting period of 10 min to allow a potential gravity-induced shift to stabilise. The 5 mm threshold was selected based on institutional workflow standards for columna deviation assessment.

For offline analysis the vertical difference in tumour position between pre- and post-treatment CBCT (δ_vrt_) were evaluated. Patients with δ_vrt_ ≥ 3 mm between pre- and post-CBCTs were flagged for intervention at the next treatment fraction. The 3 mm threshold was chosen as the largest whole number below the 4 mm PTV margin, ensuring intra-fractional drift remained within tolerance at subsequent fractions.

At the second fraction (F2), patients flagged at F1 had to wait 10–15 min in the treatment position before pre-CBCT_1_ acquisition. A post-CBCT was acquired, and the same offline assessment protocol was then followed. At the third fraction (F3), only pre-CBCTs were acquired, with waiting times of 0–20 min based on the results from previous fractions. The complete workflow diagram is provided in Supplementary Material A.

### 2.4 Image acquisition and analysis

The CBCT images were acquired with parameters: 125 kV, 268 mAs, 17.9 s acquisition time, filtered back-projection reconstruction, and 2 mm slice thickness. Online and offline matching was performed using the automatic intensity-based grey-value registration algorithm on the GTV. All position measurements used the geometric centre of the tumour. To isolate tumour-specific motion, patient displacement was subtracted (measured using bone registration) from the observed tumour displacement between pre- and post-treatment CBCTs.

Accelerator log files were analysed to determine time points from patient positioning to each CBCT acquisition. The patient couch positioning time was identified by the characteristically large longitudinal shift matching to the reference marker. Vertical tumour positions were plotted against time for F1 and F2 and fitted with linear regression to obtain a slope (mm/min) representing the average tumour drift rate, resulting in a single parameter describing vertical tumour motion. Individual patient regression analyses are shown in Supplementary Material S1.

### 2.5 Statistical analysis

Statistical analyses were performed using MATLAB 2023a (MathWorks, Natick, MA, USA) with significance level α = 0.05. Translational tumour shifts from F1 and F2 were tested using Wilcoxon signed-Rank Test (null hypothesis: median shift equals zero). A two one-sided tests (TOST) procedure evaluated whether tumour shifts were negligible, using an equivalence margin of ±1 mm based on clinical experience regarding CBCT matching precision, with t-based 90% confidence intervals.

Correlation analyses between drift slopes and patient specific parameters used Spearman correlation for continuous variables, such as age, tumour size and the tumour position in the lung. A Kruskal-Wallis test was used for lung lobe comparison, and Mann-Whitney *U* test for binary variables, such as gender assigned at birth and chronic obstructive pulmonary disease (COPD). To quantify the amplitude of respiratory-induced tumour motion IGTV_vol_/GTV_vol_ was included as a continuous variable to test whether larger breathing motion was associated with gravity-induced posterior drift. Multivariate analysis employed Principal Component Analysis (PCA) combined with linear regression.

For patients with total vertical shifts exceeding the 4 mm posterior PTV margin, clinical treatment plans were recalculated as is with rigid isocentre shifts corresponding to the maximum observed translational shifts. Dose volume histograms (DVHs) for the GTV with and without shifts were extracted from Eclipse at 0.1 Gy resolution and were compared using Wilcoxon rank sum test (null hypothesis: identical DVH distributions).

## 3 Results

**Fig. 1:**
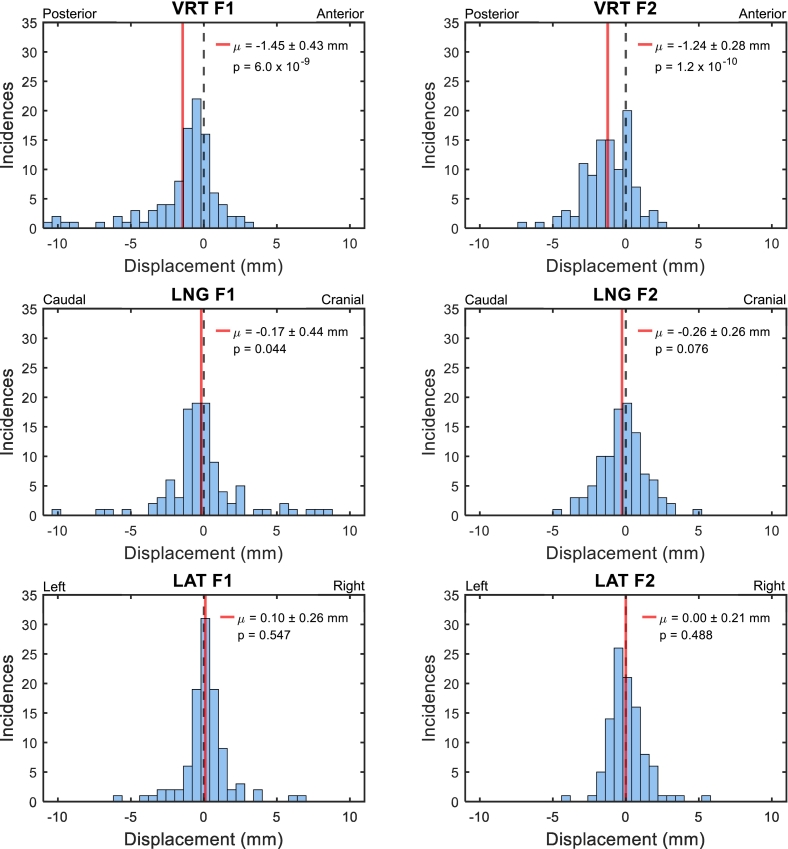
Vertical (VRT), longitudinal (LNG) and lateral (LAT) tumour shifts for F1 and F2. Zero is marked with a dashed line and the mean shift is marked with a red line, with a 90% confidence interval of the mean value. The *p*-value indicates if the distribution mean differs from zero. (For interpretation of the references to colour in this figure legend, the reader is referred to the web version of this article.)

Among the 102 tumours analysed, systematic posterior drift was observed across the cohort. The mean vertical shift and standard deviation was −1.45 ± 0.43 mm at F1 (range: −11.0 to 2.9 mm) and − 1.24 ± 0.27 mm at F2 (−7.4 to 2.4 mm), both significantly different from zero (*p* < 0.001). In contrast, lateral shifts showed no systematic pattern with mean values of 0.10 ± 0.27 mm at F1 and 0.00 ± 0.21 mm at F2. Longitudinal shifts were − 0.17 ± 0.43 mm at F1 (*p* = 0.044) and − 0.26 ± 0.25 mm at F2 (*p* = 0.076) (Fig. 1).

**Fig. 2:**
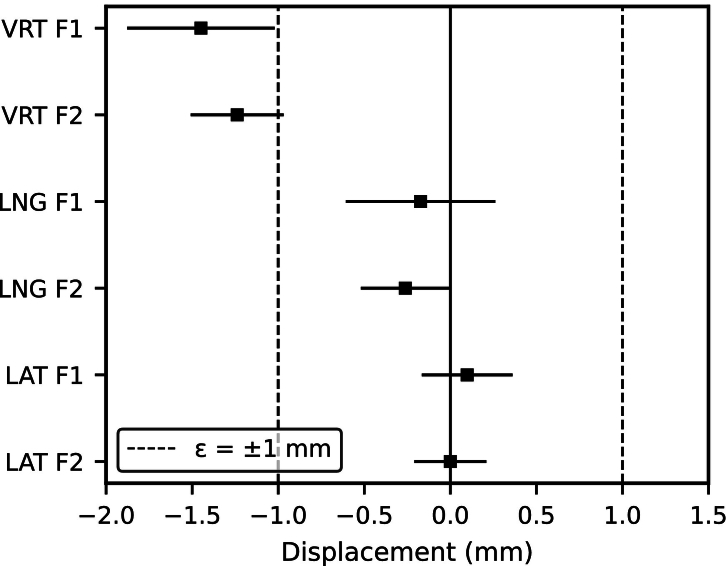
Forest plot of tumour shifts with 90% confidence intervals. The dotted lines indicate the pre-specified equivalence margin (ε = 1 mm). Shifts are shown for the vertical, longitudinal, and lateral directions for F1 and F2.

The TOST analysis demonstrated that the 90% confidence intervals for longitudinal and lateral shifts were fully contained within the ±1 mm equivalence margin for both fractions, confirming these movements as clinically negligible. Conversely, vertical shifts exceeded this margin for both fractions, indicating systematic posterior drift (Fig. 2).

The median time between pre- and post-CBCT acquisition was 14 min 22 s (range: 7:59 min 59 s to 22 min 4 s). Linear regression analysis of vertical tumour position versus time yielded patient-specific drift rates (S1). Based on these temporal drift data, 1 patient (1.0%), 16 patients (15.7%), and 28 patients (27.5%) would be expected to exceed the 4 mm PTV margin at treatment times of 10, 20, and 30 min, respectively. In total, 41 (40.2%) patients were flagged for intervention at either F1 or F2.

No significant correlations were identified between vertical drift rate and any patient or tumour specific characteristics (all *p* > 0.09, Table 1). Multivariate PCA required six components to explain 90% of the variance, indicating high dimensional complexity without dominant predictive factors.

**Table 1:** Correlation between tumour-, and patient-specific parameters and the vertical tumour drift rate where IGTVvol/GTVvol represents the respiratory-induced tumour motion.

	p-value
Tumour size	0.926
IGTV_vol_/GTV_vol_	0.168
Lung lobe	0.913
Cranio-Caudal position	0.213
Left-Right position	0.503
Anterior-Posterior position	0.096
Age	0.992
Gender	0.805
COPD	0.556

**Fig. 3:**
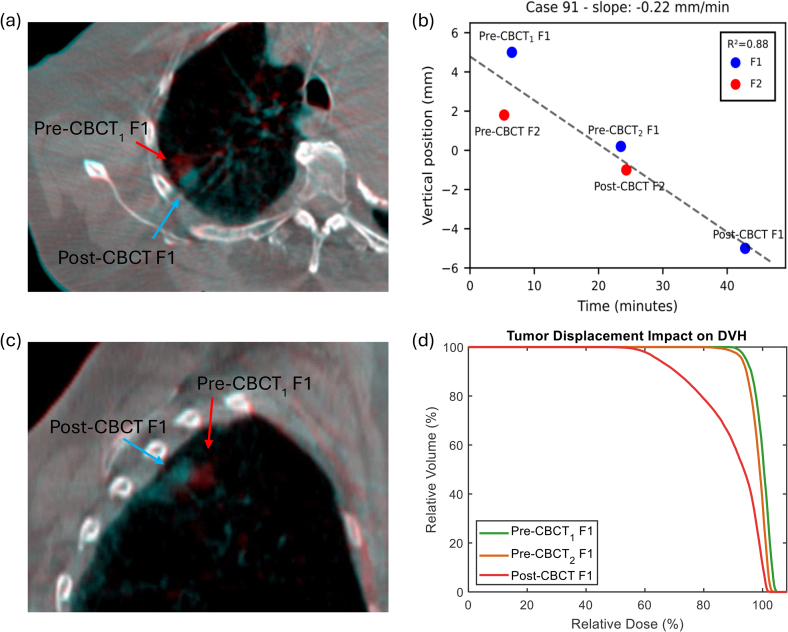
*An example with a large posterior tumour drift. (a) and (c) shows the tumour position in the transversal and sagittal view respectively, where the corresponding CT has been annotated with red indicating the first pre-CBCT and cyan indicating the post-CBCT. (b) shows the linear regression of the posterior drift and (d) shows the impact on the DVH for the GTV due to the drift*. (For interpretation of the references to colour in this figure legend, the reader is referred to the web version of this article.)

To assess worst-case scenarios in the absence of any posterior drift management, 16 patients (15.7%) with an observed total vertical drift exceeding the 4 mm PTV margin were analysed. This analysis considered the largest observed drift for each patient across both fractions, regardless of intervention. Fig. 3 illustrates a representative case (patient 91) demonstrating substantial posterior drift. The transversal and sagittal CBCT images (panels a and c) show the tumour position at pre-CBCT_1_ F1 (initial position) and post-CBCT F1 (after drift), revealing a 10 mm posterior displacement with minimal change in bony anatomy. The temporal progression (panel b) shows the linear drift pattern across both fractions, with a slope of −0.22 mm/min. The impact on GTV dose coverage (panel d) demonstrates the progressive consequences of unmanaged posterior drift: the green curve shows ideal GTV coverage if no drift occurred, the yellow curve shows slight underdosage if the tumour had drifted to the pre-CBCT_2_ position during treatment, and the red curve shows severe coverage loss if the tumour had drifted to the post-CBCT position - representing near-complete geographic miss.

**Fig. 4:**
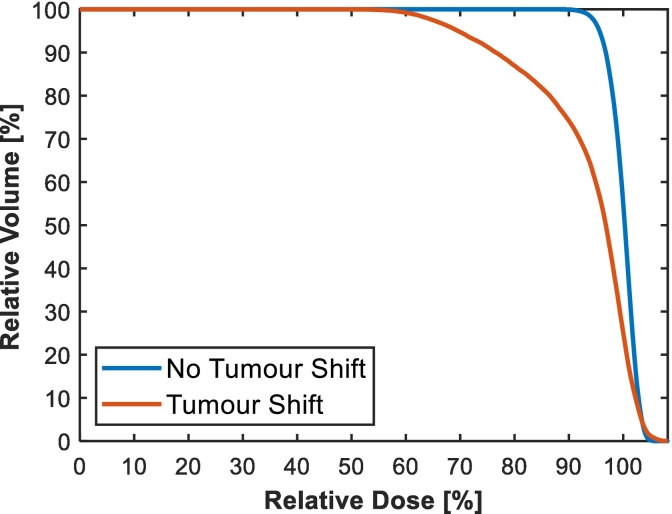
Mean GTV DVH-curve, for 16 cases with a potential worst-case posterior tumour drift larger than the PTV margin of 4 mm.

Recalculation of dose/volume metrics with worst-case isocentre shifts revealed significant GTV underdosage across all 16 affected patients (Fig. 4). The mean GTV dose decreased from 100.00 ± 0.00% without shift to 93.10 ± 5.93% with shift (*p* < 0.001). More critically, D_90%_ coverage dropped from 99.93 ± 0.15% to 74.20 ± 21.90% (p < 0.001).

## 4 Discussion

In this study, gravity-induced tumour drift was observed in free-breathing peripheral lung SABR patients using repeat CBCT imaging. Systematic posterior tumour drift was observed, with mean posterior drifts of −1.45 mm (max: −11.0 mm) at F1 and − 1.24 mm (max: −7.4 mm) at F2, whereas mean lateral and longitudinal drifts remained clinically negligible. A gravity-adapted workflow identified 41 patients (40.1%) requiring intervention and reduced extreme drift cases. Tumours with a total drift exceeding the PTV margin showed severe target underdosage in a worst-case recalculation.These findings align with and expand upon previous observations of baseline drift during thoracic radiotherapy. Guckenberger et al. reported mean three-dimensional intra-fractional tumour drift relative to bony anatomy of 2.3 ± 1.6 mm (maximum 7.4 mm) with a systematic posterior component of 0.8 mm in free-breathing SABR [10]. This study revealed larger systematic posterior components (1.45 mm), which may indicate that gravity's contribution is more substantial than previously recognised, although differences between patient cohorts could also explain the discrepancy.

Studies utilising fiducial markers placed in lung lymph nodes or adjacent to tumours, as a live tumour tracking surrogate, showed a systematic posterior and cranial drift during lung treatment in free breathing [9], [15]. Takao et al. documented baseline drifts >3 mm in 71.6% of fractions within 30 min [9]. Similarly, Seppenwoolde et al. specifically noted posterior trends during respiratory-gated treatments, attributing them to patient relaxation and gravity acting on compliant lung tissue [8]. These studies align with the observations of a posterior drift seen in this study, supporting evidence that tumour position drifts with time.

Posterior drift is not only limited to lung tissue, Fast et al. observed posterior liver drift reaching 5 mm over 40 min on cine-magnetic resonance imaging (MRI) [16]. Similarly, Grimbergen et al. reported baseline drifts requiring intervention in upper abdomen treatments [17], [18]. The consistency of posterior drift across different organs and treatment techniques suggests a common gravitational mechanism affecting compliant tissues in supine positioning.

A recent multi-institutional audit by Rough et al. found small mean shifts, but occasional outliers >3 mm in both lung and liver treatment between 5 and 25 min, emphasizing the need for intra-fraction imaging to identify clinically relevant cases [19]. The data of this study strongly support this conclusion, with 15.7% of patients exhibiting posterior drifts exceeding the PTV margin despite acceptable population means.

The use of linear regression to characterise drift rates represented a deliberate simplification of what is likely a more complex temporal pattern. Although an exponential decay model would theoretically better represent viscoelastic relaxation approaching equilibrium, the linear model provided a single patient-specific parameter suitable for correlation analysis and visual interpretation as seen in Supplementary Material S1. Constructing an exponential decay model for each patient would require more tumour position data points (i.e., additional CBCT scans), thereby increasing imaging dose without evidence of clinical benefit and rendering this approach infeasible. Importantly, the success of the waiting time interventions implicitly acknowledges non-linear drift behaviour. The diverse patient-specific patterns visible in Supplementary Material S1, with some patients appearing to plateau while others show continued drift, underscore the complexity of individual response patterns and justify our patient-specific management approach.

The observed posterior drift aligns with established lung physiology and biomechanics. Evidence from MRI- and CT-based analyses has shown position-dependent redistribution of stress within lung tissue, confirming the role of gravity in lung deformation [12], [13]. Critically, lung parenchyma exhibited viscoelastic properties with time-dependent deformation under sustained loading [14], providing the mechanistic basis for the slow, monotonic drift we observed. While factors such as muscular or diaphragmatic relaxation cannot be excluded, the consistency of the posterior direction and the monotonic nature of the drift suggest that parenchymal compliance and gravity-driven deformation are the primary drivers.

The absence of correlation between patient or tumour parameters and tumour drift suggests that gravitational effects on lung compliance are universal rather than restricted to specific patient subgroups. While factors such as COPD might theoretically alter tissue compliance, the results indicate that all patients required assessment for potential drift.

The tumour dose coverage analysis from both the individual case example and the cohort analysis revealed severe consequences for unmanaged drift. For patients with a tumour drift larger than the posterior PTV-margin the average worst-case D_90%_ coverage dropped to 74.2%. Given SABR's reliance on steep dose gradients for efficacy, such underdosing could significantly compromise local control, underscoring the clinical importance of identifying and managing gravity-induced drift in free-breathing lung SABR. The results of this study suggest three management approaches:

First, our patient-specific workflow using pre- and post-treatment CBCT with selective waiting periods successfully managed all cases including extreme drifts. This approach avoided unnecessary interventions for the ∼85% of patients without significant drift while ensuring safety for those at risk. Second, institutions could implement universal waiting periods before initial imaging, likely stabilising most patients, but potentially insufficient for extreme cases. Third, posterior PTV margin expansion—meaning an approximate increase from 4 mm to 5 mm, could accommodate most drift, though this simplified approach ignores the time-dependent nature of the phenomenon and unnecessarily enlarges treatment volumes for patients without drift.

Treatment time emerged as a critical factor: reducing CBCT matching and treatment delivery from 20 to 10 min would decrease affected patients from 15.7% to 1.0%. The average time between Pre- and Post-CBCT in this study was 14 min 22 s, which is comparable to other studies using similar treatment modalities [19], [20]. Enabling faster delivery may inherently mitigate drift risk, alternatively technologies such as real-time kV tracking or mid-treatment CBCT could also identify and correct drift during delivery [21], [22].

The present study has several limitations. The TOST analysis used an equivalence margin (ε = 1 mm) based on clinical judgement and experience with CBCT matching rather than a statistical or population-based definition, and the results are therefore conditional on this assumption. In addition, the single-institution design limits generalisability, although the underlying physiological mechanism may be broadly applicable.

Future research should investigate optimal waiting times through more detailed temporal analysis. Development of predictive models using patient and tumour specific parameters, despite the negative findings of this study, might benefit from larger multi-institutional datasets. Long-term outcome analysis correlating drift management with local control would strengthen the clinical rationale for systematic monitoring.

In conclusion, this study provided the first systematic evidence of gravity-induced posterior tumour drift in free-breathing peripheral lung SABR, with 15.7% of patients experiencing drift exceeding PTV margins. The phenomenon appeared universal rather than limited to specific patient subgroups, emphasizing the need for systematic assessment for all patients. Our gravity-adapted workflow successfully identified and managed at-risk patients while avoiding unnecessary interventions for the majority. The severe consequences for tumour dose coverage observed underscore the clinical importance of this previously under-recognised phenomenon. We recommend that all institutions treating lung SABR implement at minimum post-treatment CBCT verification to identify patients with significant drifts, enabling appropriate management strategies for subsequent fractions.

## CRediT authorship contribution statement

**Lars Merring-Mikkelsen:** Writing – original draft, Visualization, Project administration, Investigation, Formal analysis, Data curation, Conceptualization. **Hella M.B. Sand:** Writing – review & editing, Project administration, Conceptualization. **Mads H. Brincker:** Writing – original draft, Visualization, Project administration, Investigation, Formal analysis, Data curation, Conceptualization.

## Declaration of competing interest

The authors declare that they have no known competing financial interests or personal relationships that could have appeared to influence the work reported in this paper.
