# Supplementary material for: Systematic gravity-induced posterior tumour drift in free-breathing lung stereotactic ablative radiotherapy
Source: Phys Imaging Radiat Oncol. 2026 Jul 6;40:101030. doi: 10.1016/j.phro.2026.101030 (PMC13382430; doi:10.1016/j.phro.2026.101030)
Supplement: Supplementary file 1 — Supplementary material [file mmc1.pdf]

Supplementary Material A:

SBRT gravity workflow

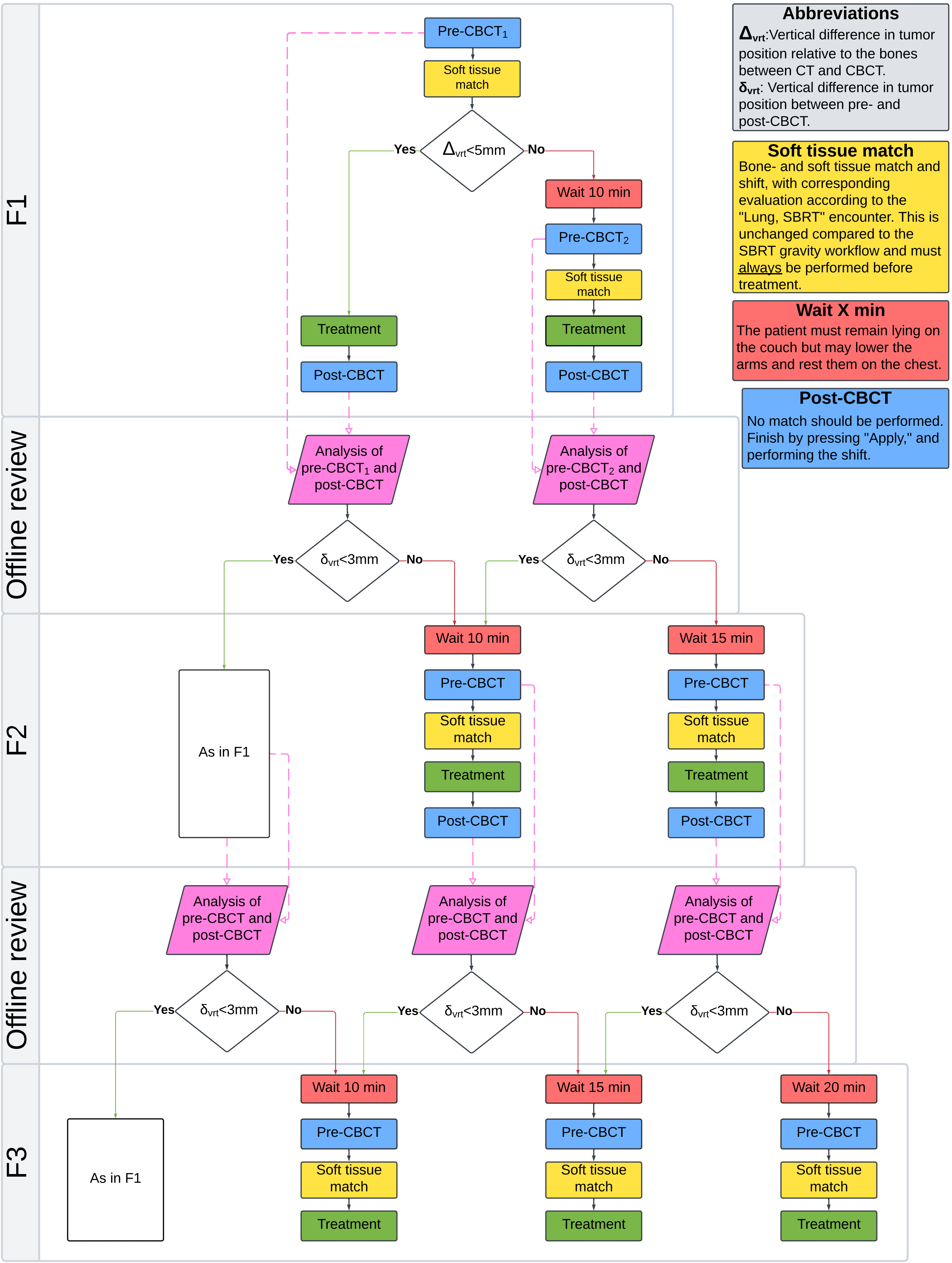

Abbreviations

Δ_{vrt}:Vertical difference in tumor position relative to the bones between CT and CBCT.
δ_{vrt}: Vertical difference in tumor position between pre- and post-CBCT.

Soft tissue match

Bone- and soft tissue match and shift, with corresponding evaluation according to the "Lung, SBRT" encounter. This is unchanged compared to the SBRT gravity workflow and must always be performed before treatment.

Wait X min

The patient must remain lying on the couch but may lower the arms and rest them on the chest.

Post-CBCT

No match should be performed. Finish by pressing "Apply," and performing the shift.

Supplementary Material S1

Fig. S1.1: Case-specific linear regressions of vertical tumour drift during SABR. Blue and red markers denote measurements from the first fraction (F1) and second fraction (F2), respectively. Dashed black lines indicate regression fits; slopes (mm/min) and coefficients of determination (R^2) are reported in each subplot. Vertical positions were offset such that the first F1 measurement equals zero, providing uniform scaling across patients without affecting slope estimation. Subplots are ordered by increasing slope, illustrating inter-patient variability and a systematic posterior drift. The y-axis shows tumour vertical position (mm), and the x-axis shows elapsed time (minutes) from patient setup on the treatment couch. The figure extends across to the next page.

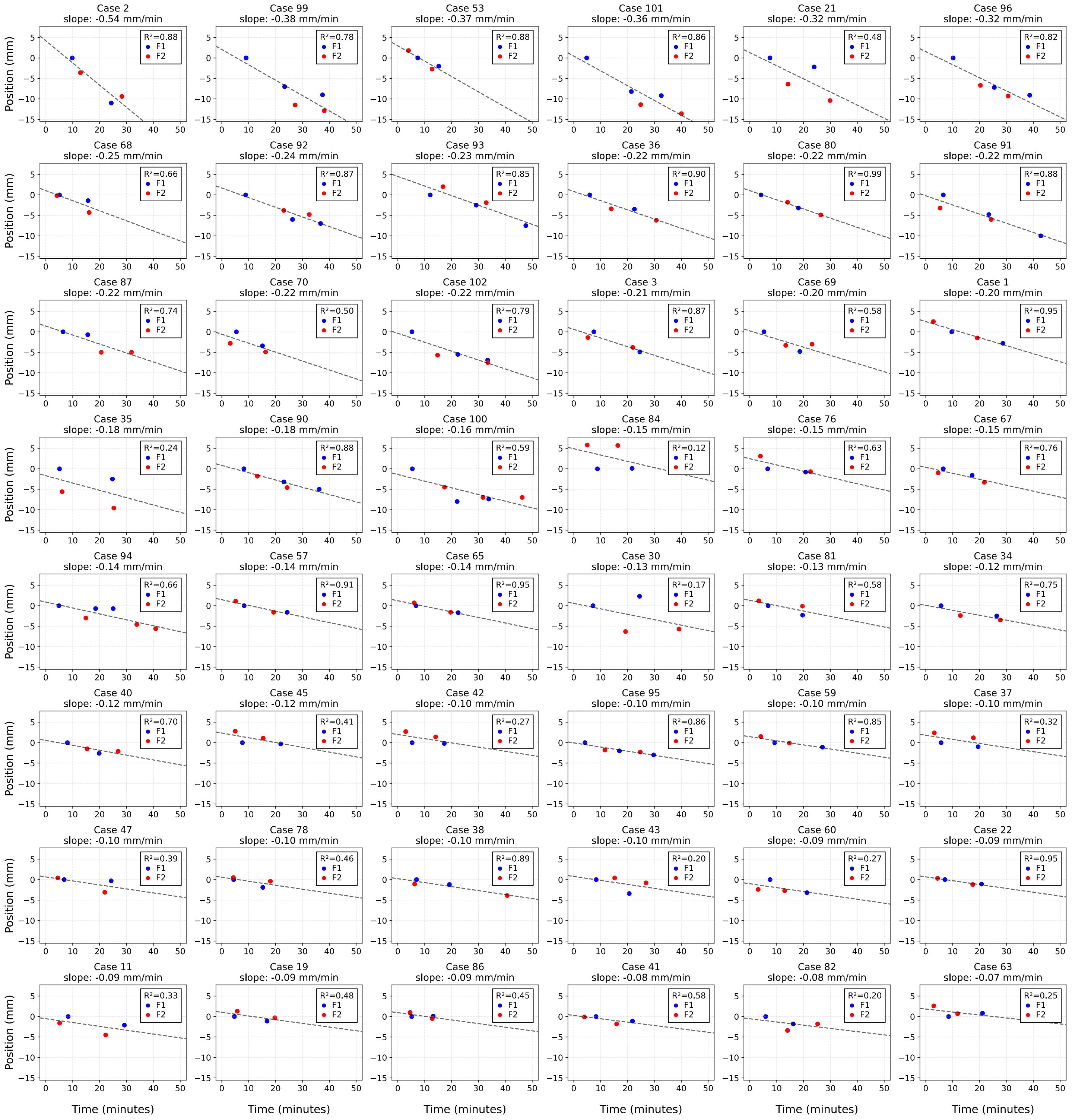

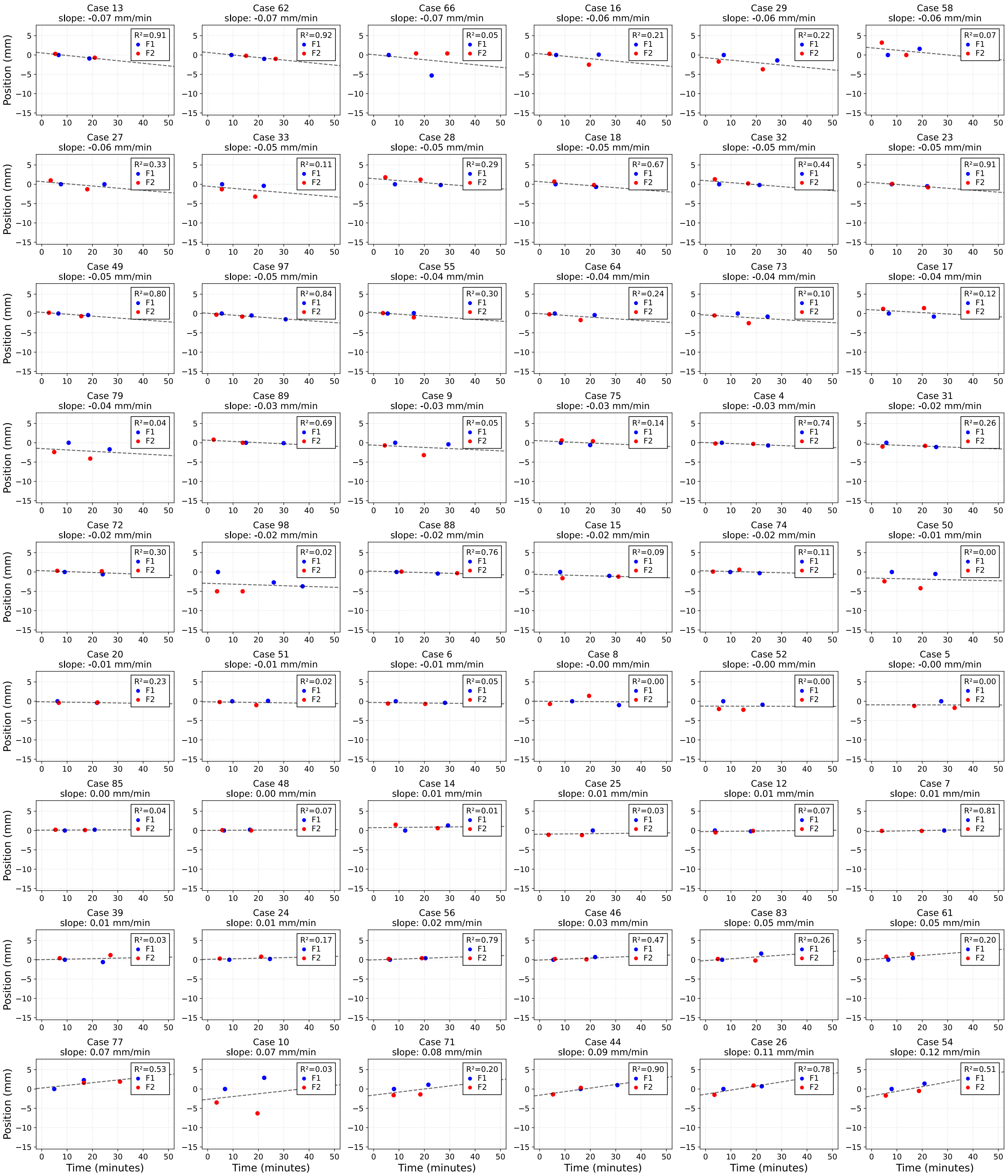
Case 13
slope: -0.07 mm/min
R²=0.91
Case 62
slope: -0.07 mm/min
R²=0.92
Case 66
slope: -0.07 mm/min
R²=0.05
Case 16
slope: -0.06 mm/min
R²=0.21
Case 29
slope: -0.06 mm/min
R²=0.22
Case 58
slope: -0.06 mm/min
R²=0.07
Case 27
slope: -0.06 mm/min
R²=0.33
Case 33
slope: -0.05 mm/min
R²=0.11
Case 28
slope: -0.05 mm/min
R²=0.29
Case 18
slope: -0.05 mm/min
R²=0.67
Case 32
slope: -0.05 mm/min
R²=0.44
Case 23
slope: -0.05 mm/min
R²=0.91
Case 49
slope: -0.05 mm/min
R²=0.80
Case 97
slope: -0.05 mm/min
R²=0.84
Case 55
slope: -0.04 mm/min
R²=0.30
Case 64
slope: -0.04 mm/min
R²=0.24
Case 73
slope: -0.04 mm/min
R²=0.10
Case 17
slope: -0.04 mm/min
R²=0.12
Case 79
slope: -0.04 mm/min
R²=0.04
Case 89
slope: -0.03 mm/min
R²=0.69
Case 9
slope: -0.03 mm/min
R²=0.05
Case 75
slope: -0.03 mm/min
R²=0.14
Case 4
slope: -0.03 mm/min
R²=0.74
Case 31
slope: -0.02 mm/min
R²=0.26
Case 72
slope: -0.02 mm/min
R²=0.30
Case 98
slope: -0.02 mm/min
R²=0.02
Case 88
slope: -0.02 mm/min
R²=0.76
Case 15
slope: -0.02 mm/min
R²=0.09
Case 74
slope: -0.02 mm/min
R²=0.11
Case 50
slope: -0.01 mm/min
R²=0.00
Case 20
slope: -0.01 mm/min
R²=0.23
Case 51
slope: -0.01 mm/min
R²=0.02
Case 6
slope: -0.01 mm/min
R²=0.05
Case 8
slope: -0.00 mm/min
R²=0.00
Case 52
slope: -0.00 mm/min
R²=0.00
Case 5
slope: -0.00 mm/min
R²=0.00
Case 85
slope: 0.00 mm/min
R²=0.04
Case 48
slope: 0.00 mm/min
R²=0.07
Case 14
slope: 0.01 mm/min
R²=0.01
Case 25
slope: 0.01 mm/min
R²=0.03
Case 12
slope: 0.01 mm/min
R²=0.07
Case 7
slope: 0.01 mm/min
R²=0.81
Case 39
slope: 0.01 mm/min
R²=0.03
Case 24
slope: 0.01 mm/min
R²=0.17
Case 56
slope: 0.02 mm/min
R²=0.79
Case 46
slope: 0.03 mm/min
R²=0.47
Case 83
slope: 0.05 mm/min
R²=0.26
Case 61
slope: 0.05 mm/min
R²=0.20
Case 77
slope: 0.07 mm/min
R²=0.53
Case 10
slope: 0.07 mm/min
R²=0.03
Case 71
slope: 0.08 mm/min
R²=0.20
Case 44
slope: 0.09 mm/min
R²=0.90
Case 26
slope: 0.11 mm/min
R²=0.78
Case 54
slope: 0.12 mm/min
R²=0.51
F1
F2
Position (mm)
Time (minutes)
